# Integrative Epigenome Map of the Normal Human Prostate Provides Insights Into Prostate Cancer Predisposition

**DOI:** 10.3389/fcell.2021.723676

**Published:** 2021-08-26

**Authors:** Tao Wang, Juan Song, Min Qu, Xu Gao, Wenhui Zhang, Ziwei Wang, Lin Zhao, Yan Wang, Bing Li, Jing Li, Jinjian Yang

**Affiliations:** ^1^Department of Urology, The First Affiliated Hospital of Zhengzhou University, Zhengzhou University, Zhengzhou, China; ^2^Shanghai Key Laboratory for Tumor Microenvironment and Inflammation, Department of Biochemistry and Molecular Cell Biology, Shanghai Jiao Tong University School of Medicine, Shanghai, China; ^3^Department of Urology, Changhai Hospital, Second Military Medical University, Shanghai, China; ^4^Department of Bioinformatics, Center for Translational Medicine, Second Military Medical University, Shanghai, China

**Keywords:** epigenetics, prostate, histone modification, ChIP-seq, WGBS, ATAC-seq, RNA-seq

## Abstract

Cells of all tissues in the human body share almost the exact same DNA sequence, but the epigenomic landscape can be drastically distinct. To improve our understanding of the epigenetic abnormalities in prostate-related diseases, it is important to use the epigenome of normal prostate as a reference. Although previous efforts have provided critical insights into the genetic and transcriptomic features of the normal prostate, a comprehensive epigenome map has been lacking. To address this need, we conducted a Roadmap Epigenomics legacy project integrating six histone marks (H3K4me1, H3K4me3, H3K9me3, H3K36me3, H3K27me3, and H3K27ac) with complete DNA methylome, transcriptome, and chromatin accessibility data to produce a comprehensive epigenome map of normal prostate tissue. Our epigenome map is composed of 18 chromatin states each with unique signatures of DNA methylation, chromatin accessibility, and gene expression. This map provides a high-resolution comprehensive annotation of regulatory regions of the prostate, including 105,593 enhancer and 70,481 promoter elements, which account for 5.3% of the genome. By comparing with other epigenomes, we identified 7,580 prostate-specific active enhancers associated with prostate development. Epigenomic annotation of GWAS SNPs associated with prostate cancers revealed that two out of nine SNPs within prostate enhancer regions destroyed putative androgen receptor (AR) binding motif. A notable SNP rs17694493, might decouple AR’s repressive effect on CDKN2B-AS1 and cell cycle regulation, thereby playing a causal role in predisposing cancer risk. The comprehensive epigenome map of the prostate is valuable for investigating prostate-related diseases.

## Introduction

In the 1940s, the term epigenetics was first introduced to describe the interaction between a phenotype and the environment (Waddington, 2012). Interest in epigenetics has been fueled by accumulating evidence that the mechanisms are associated with various human diseases and developmental stages. This includes nearly all types of cancer, and autoimmune, cardiovascular, and hereditary disorders (van der Harst et al., 2017; Cavalli and Heard, 2019). The Human Genome Project provided a high-quality human genome assembly, a milestone in genomic and medical research (Collins et al., 2003). Although all cells and tissues in the human body share a nearly identical genome, the epigenomic landscape varies as a function of cell type, developmental stage, and environmental impact. To annotate regulatory regions of the genome, large-scale mapping of epigenomic modifications has been undertaken in recent years. The NIH Roadmap Epigenomics Consortium integrated epigenomic maps to develop a database of tissue-specific functional elements, with distinct chromatin states and generated reference epigenomes for 127 human tissues and primary cells (Roadmap Epigenomics Consortium et al., 2015). These maps have been extensively used to gain an in-depth understanding of the role of epigenomic modifications underlying diverse human traits, as well as gene regulation, cell differentiation, and tumorigenesis (Chen et al., 2016; Yu et al., 2016; Pomerantz et al., 2020). However, prostate tissues were not included in these initial studies.

The prostate gland is a male reproductive organ that produces seminal fluids to feed and protect sperm cells. It is also associated with hormone release and sexual health (Verze et al., 2016). On the contrary, adenocarcinoma of the prostate is one of the leading causes of cancer-related deaths among men (Cornford et al., 2021). Most studies have confirmed that prostate cancer is often associated with a variety of abnormal epigenetic modifications of the genome, such as the global loss of DNA methylation, reprogramming of histone modification marks, and abnormal activation of tissue-specific enhancers, among others (Stelloo et al., 2018; Zhao et al., 2020). To gain a clear understanding of the epigenetic abnormalities in prostate cancer, it is important to use the epigenome, including detailed maps of *cis*-regulatory elements and chromatin states, of the normal prostate tissue as a reference. However, the majority of epigenomic datasets available are from prostate cancer, instead of normal prostate tissue (Stelloo et al., 2018; Rhie et al., 2019).

To close this knowledge gap, we produced multiple omics datasets from the normal prostate, including histone modifications, DNA methylation, chromatin accessibility, and RNA transcripts. We generated a high-resolution reference epigenome map to facilitate investigation of the normal biology and pathophysiology of the prostate. These annotations were used to identify epigenome differences between the prostate and other tissues. Furthermore, by comparing with previously published Roadmap epigenomes, we defined prostate-specific regulatory elements and made these resources publicly and freely available. By applying the prostate reference epigenome to functionally annotate genetic variants associated with prostate cancer, we identified two GWAS SNPs in prostate enhancers that may disrupt androgen receptor (AR) binding and the target gene regulatory network, thereby providing a mechanistic hypothesis regarding genetic predisposition for the disease.

## Materials and Methods

### Sample Collection

Prostate specimens were collected from radical cystectomies treating bladder cancer at the Urology Department of Changhai Hospital, Shanghai, China. Informed assent/consent was obtained in accordance with Chinese legislation. Ethical committee approval was secured from Changhai Hospital (CHEC2019-012). All samples were immediately frozen after collection in liquid nitrogen and stored at –80°C. Hematoxylin and eosin-stained (H&E) slides from each case were independently reviewed by two genitourinary pathologists. Samples enriched with normal prostate epithelium (> 70%) were used for the analyses. The clinical information of all cases is presented in Supplementary Table 1.

### DNA and RNA Extraction

Genomic DNA was extracted using the DNeasy Tissue Kit (Qiagen) according to the manufacturer’s protocol. RNA was extracted using TRIzol reagent (Invitrogen). The total DNA/RNA concentration was measured using a Qubit fluorometer (Invitrogen). RNA purity was checked using a NanoPhotometer spectrophotometer (IMPLEN, München, Germany).

### Chromatin Immunoprecipitation Sequencing (ChIP-Seq) Library Generation

Samples were cut into 2–3 mm 3 pieces, fixed in 1.5% formaldehyde for 10 min, and quenched with glycine. The tissues were mechanically extracted by applying 50–75 strokes using a Dounce homogenizer (Type B). Chromatin was sheared to 200–500 bp using a high-power Bioruptor Plus sonicator for 30 cycles (10 s ON, 10 s OFF). For each ChIP, 1–3 μg of antibodies were conjugated with 100 μL Protein G Dynabeads (Thermo Fisher Scientific, Cat. No. 10004D, 5 mL). Antibodies against the histone marks H3K4me3 (ab8580, Abcam), H3K4me1 (ab8895, Abcam), H3K9me3 (ab8898, Abcam), H3K36me3 (ab9050, Abcam), H3K27ac (ab177178, Abcam), and H3K27me3 (Cat. No: 39155, Active Motif) were used for immunoprecipitation. The immunoprecipitated and input DNA was purified with QIAquick Spin Columns (QIAGEN) and then subjected to library preparation using the ThruPLEX DNA-seq 48D Kit (Rubicon Genomics) according to the manufacturer’s instructions. The libraries were inspected with a Qubit fluorometer, Agilent Bioanalyzer 2100 system, and StepOnePlus Real-Time PCR.

### mRNA Sequencing (RNA-Seq) Library Generation

The RNA-seq libraries were generated using the NEBNext Ultra^TM^ RNA Library Prep Kit for Illumina (NEB, United States) according to the manufacturer’s protocol. In total, 3 μg RNA was used for each sample. Briefly, mRNA was purified using oligo (dT) magnetic beads. Purified RNA was subjected to fragmentation, reverse transcription, end-repair, 3’-end adenylation, adaptor ligation, and polymerase chain reaction (PCR) amplification. The final product was purified using the AMPure XP system, and library quality was checked using the Agilent Bioanalyzer 2100 system.

### Assay for Transpose Accessible Chromatin Using Sequencing (ATAC-Seq) Library Generation

The fresh-frozen samples were disassociated as previously described (Corces et al., 2017). A total of 50,000 isolated nuclei were used, and library preparation was performed using the Nextera DNA Library Preparation Kit (Illumina) according to the manufacturer’s protocol. Transposed DNA was then purified using the MinElute PCR Purification Kit (Qiagen), amplified using the NEBNext High-Fidelity PCR Master Mix (New England Biolabs, Ipswish, MA, United States), and purified using the MinElute PCR Purification Kit (Qiagen).

### Whole-Genome Bisulfite Sequencing (WGBS) Library Generation

In total, 5.2 μg of genomic DNA and 26 ng lambda DNA were sheared to 200–500 bp using a Bioruptor Plus sonicator. Cytosine-methylated barcodes were ligated to DNA fragments. Lambda DNA was used to calculate the bisulfite conversion rate. These DNA fragments were treated twice with bisulfite using the EZ DNA Methylation-Gold Kit (Zymo Research) according to the manufacturer’s instructions. Subsequently, the single-strand DNA was PCR-amplified using KAPA HiFi HotStart Uracil + ReadyMix (2X), and the insert size was assayed on an Agilent Bioanalyzer 2100 system.

### Generation of Sequencing Data

All libraries were subjected to sequencing on the Illumina NovaSeq 6000 platform, and 150 bp paired-end reads were generated. FastQC (v.0.11.8)^1^ was used to assess quality of the raw reads. The reads were pre-processed using Trimmomatic (v.0.39) (Bolger et al., 2014) using the following parameters: LEADING:3 TRAILING:3 adapter.fa:2:30:10 SLIDINGWINDOW:4:15 MINLEN:36. The clean reads that passed all the filtering steps were used for downstream analyses.

### ChIP-Seq Processing

Clean reads were mapped to hg19 using BWA-MEM (v.0.7.17) (Li and Durbin, 2009). Multiple-mapped reads were filtered using Samtools (v.1.9) (Li et al., 2009), and PCR-duplicated reads were removed using Picard.^2^ Index of the bam files was generated using Samtools. The overall quality control of the ChIP-seq data was evaluated using the ChIPQC R package (v.4.0.2) (Carroll et al., 2014; Supplementary Table 1). To examine the reproducibility of the ChIP-seq experiments, correlation coefficients were calculated between replicates using the read coverages of 10 kb-binned matrices using deepTools 2.0 (Ramirez et al., 2016). DeepTools was also used to plot the gene body and flanking region heatmap graphs using the normalized signal intensity. The ChIP-seq signals over the input background were visualized on the WashU Epigenome Browser using the MACS2 (Zhang et al., 2008) bdgcmp function with the following parameter: -m FE. The MACS2 peak caller was used to identify narrow peaks for H3K4me3, and H3K27ac using a *q*-value threshold of 0.05 and broad domains for H3K4me1, H3K36me3, H3K9me3, and H3K27me3 using a *q*-value threshold of 0.1.

### ATAC-Seq Processing

The clean reads were mapped to hg19, and the aligned reads were filtered in the same way as the ChIP-seq data processing. Reads mapped to blacklist regions or mitochondria were removed. All filtered reads mapped to the positive strand were offset by + 4 bp, and reads mapped to the negative strand were offset by -5 bp to reflect the actual binding sites of transposons using deepTools with the following command: Alignmentsieve –ATACshift. The Spearman correlation coefficient was calculated between replicates (Supplementary Table 1), and signals were calculated for visualization, similar to ChIP-seq. To evaluate the chromatin accessibility of each state, we calculated the -log10 (*p*-value) scores using the MACS2 bdgcmp function with the following parameter: -m ppois. The MACS2 peak caller was used to identify narrow ATAC peaks using a *q*-value threshold of 0.05. The peaks were merged to create a union set of sites. All merged peaks were separated into proximal ATAC-seq peaks (*n* = 13,553), which were defined as overlapping with promoters [regions as 2 kb upstream and 500 bp downstream of transcription start site (TSS)], and distal ATAC-seq peaks (*n* = 27,840) (Supplementary Table 2).

### RNA-Seq Processing

Clean reads were mapped to hg19 using STAR (v.2.7.6a) (Dobin et al., 2013). Multiple-mapped reads were then removed, and the correlation coefficient was calculated between replicates (Supplementary Table 1), and normalized signals were calculated for visualization, similar to ChIP-seq. Filtered reads were assembled using StringTie (v.2.1.4) (Kovaka et al., 2019). Transcripts per million (TPM) were calculated for each gene. Genes were defined using the GENCODE release 29 (Harrow et al., 2012). We divided the genes into expressed and repressed prostate genes using a Gaussian mixture model. The R package mixtools (v.1.2.0) was used to perform this analysis (Scrucca et al., 2016). First, the average expression values (TPMs) of all protein-coding genes of the 3 samples in this study were taken as input. All genes were divided into 2 (*k* = 2) density functions of Gaussian distribution. In this manner, each gene was assigned to a Gaussian distribution model and received a posterior probability value. These genes defined as either expressed or repressed genes, respectively, using the cutoff value of the posterior probability of 0.9.

### WGBS Processing

The clean reads were mapped to hg19 using Bismark (v.0.22.1) (Krueger and Andrews, 2011) with the following parameters: Bowtie2 –dovetail –score_min L,0,-0.2 –nucleotide_coverage. Duplicate reads from PCR amplification were removed using the deduplicatebismark command. Cytosine methylation levels were extracted from the de-duplicated reads using the bismark_methylation_extractor command from Bismark with the following parameters: –comprehensive –ignore_r2 18 –ignore 2 –bedGraph –no_overlap –report. The Coverage2cytosine command was used to calculate the methylation and total read counts per CpG. CpGs with coverage of at least five were used for downstream analyses. The bedgraph files generated by Bismark were converted to bigwig files, which were used for visualization using BedGraphToBigWig.

### Gene Expression Omnibus (GEO) Data

The ChIP-seq data of H3K27ac with three biological replicates, FOXA1, AR, and HOXB13, and two biological replicates from NCBI’s GEO with GSE numbers GSE130408, GSE130408, and GSE70079 were downloaded from the SRA Toolkit (v.2.10.7).^3^ The downstream analysis of these datasets resembled the ChIP-seq data in this study.

### Construction of Prostate Epigenome

We applied ChromHMM (v.1.22) (Ernst and Kellis, 2012), which is based on a multivariate hidden Markov model, to compute genome-wide 15 chromatin states using five histone marks (H3K4me1, H3K4me3, H3K36me3, H3K9me3, and H3K27me3) and 18 chromatin states using six histone marks (plus H3K27ac). For each one, read counts were calculated in non-overlapping 200-bp bins across the whole genome. Each bin was assigned 0 (no signal) or 1 (signal) using the BinarizeBam command with the input alignment files as the control. The joint models, which were trained by Roadmap using 60 (for 15-state) or 40 (for 18-state) epigenomes with the highest-quality data from diverse tissues and cell types, were applied to generate those states using the MakeSegmentation command. Enrichments for annotations, including all types of genomic features, TSS/TSS neighborhood, conserved GERP elements,^4^ LMRs/UMRs, distal/proximal ATAC peaks, and FOXA1/AR/HOXB13 peaks for the 18-state or 15-state model, were computed using the OverlapEnrichment command of ChromHMM. In this study, we also created an individual model using ChIP-seq datasets of the prostate.

### Clustering Analysis and Identification of Prostate-Specific Active Enhancers

First, we downloaded the 18 states of 98 epigenomes from the Roadmap Epigenomics Project.^5^ We then extracted and merged all active enhancer states (EnhA1 and EnhA2) of the prostate and 98 epigenomes. All active enhancers were divided into non-overlapping 200-bp bins. For each tissue or cell type, each bin was assigned 0 (no enhancer) or 1 (enhancer). From this data matrix, we identified all prostate-specific enhancer bins, none of which were active enhancers in any of the other 98 epigenomes. These bins were merged to produce 7,580 prostate-specific active enhancers. The matrix was also used to calculate the pair-wise Pearson correlation coefficients among all 99 reference epigenomes. We then performed complete-linkage hierarchical clustering according to the resulting correlation matrix using the factoextra R package (v.1.0.7). We compared the active enhancers between the prostate and six other tissues or cell types as an example (E003, E034, E090, E091, E072, and E104). All active enhancer states of the samples were merged into a union set of regions. We calculated the read counts, which were then normalized to obtain reads per kilobase per million (RPKM) values of H3K27ac in these regions for each sample. We clustered all the regions into nine clusters based on the normalized intensity of H3K27ac using the k-means algorithm. Normalized H3K4me1 intensity for seven samples and ATAC-seq intensity for five samples in the corresponding enhancer clusters are also shown. GO term analysis of the top 1,000 prostate-specific active enhancers (ranked by intensity) was performed using GREAT (v.4.0.4) (McLean et al., 2010). Motif analysis of prostate-specific active enhancers was performed using HOMER2 (v.4.11) (Heinz et al., 2010).

### Identification of Prostate-Specific Genes

To identify prostate-specific genes, we used the algorithm described by the Human Protein Atlas (HPA) (Uhlen et al., 2015) and obtained 120 genes from the HPA website.^6^ We filtered these genes (TPM > 1 in our RNA-seq data) and defined 103 prostate-specific genes (Supplementary Table 3), including the following three groups: (1) prostate-enriched genes with at least four-fold higher mRNA levels in the prostate compared to any other tissue; (2) group-enriched genes with at least fourfold higher average mRNA levels in a group of 2–5 tissues, including the prostate, compared to any other tissues; (3) prostate-enhanced genes with at least fourfold higher mRNA levels in the prostate compared to the average level in all other tissues. The other 55 human tissue transcriptomes were downloaded from the GTEx Consortium. To remove the batch effect between our and the public RNA-seq libraries of normal prostate samples, we used the limma R package (v.3.44.3). First, we downloaded the gene expression value (TPMs) matrix of multiple human normal tissues used for the GTEx project from the website.^7^ The gene expression values (TPMs) of three cases of normal prostate tissue in this study and prostate tissue in GTEx project were integrated into a matrix. The data on genes whose TPM expression value was less than 1 in all samples were removed, and then log2 (TPM + 0.01) conversion was performed for all genes. The removeBatchEffect function in the limma package was used to remove the batch effect using the default parameters. After removing the batch effect, we normalized the expression data using the quantile method, and the normalized data were used for subsequent analysis. A heatmap was used to show the expression of these 103 genes in all tissues using the pheatmap R package (v.1.0.12).

### Identification of Unmethylated Regions (UMRs) and Lowly Methylated Regions (LMRs)

LMRs and unmethylated regions (UMRs) were identified for all samples using the MethylSeekR package (v.1.28.0) for R (Burger et al., 2013). First, partially methylated domains (PMDs) were identified and masked. We then ran MethylSeekR with default parameters: A coverage cutoff of 5 reads per CpG, at least 5 or 6 CpGs, FDRs of less than 0.05, and methylation level threshold set at 0.5.

### Analysis of GWAS SNPs in Tissue-Associated Enhancers

To evaluate the enrichment of SNPs in enhancers, we adopted a previously described method (Ernst et al., 2011; Roadmap Epigenomics Consortium et al., 2015). Firstly, we obtained the NHGRI GWAS catalog through the UCSC Table Browser on April 23, 2021. The enrichment of GWAS SNPs for 99 epigenome references was calculated. We excluded chromosome Y but retained chromosome X for the enrichment analysis. To reduce dependencies between pairs of SNPs assigned to the same study, we pruned SNPs such that no two SNPs were within 1 Mb of each other on the same chromosome. The pruning procedure considered each SNP in the order of their genomic locations. We retained an SNP if there was not another SNP already retained within 1 Mb. We restricted our analysis to studies reporting two or more associated SNPs. The variants from each study were intersected with active enhancer states (states 9 and 10 for the 18-state model) of each of the cell type. Hypergeometric *P*-values for the enrichment of each pruned set of SNPs overlapping enhancer states were computed against the pruned GWAS catalog as the background. We obtained the location information of SNPs from the SNPlocs.Hsapiens.dbSNP144.GRCh37 database. Functional annotation of the GWAS SNPs was performed using motifbreakR Tool (Coetzee et al., 2015) by examining a 2-kb region centered on the SNP. We used the database for *Homo sapiens* and selected the method “ic” to calculate position probability matrix (PPM). The gain or loss of the motifs around nine prostate cancer-associated GWAS SNPs was predicted using a *p*-value cutoff of 1e-04 and presented in Supplementary Table 7. For the same transcription binding sites from different database, we chose the most recent versions. The germline information was obtained from the Chinese Prostate Genome and Epigenome Atlas (CPGEA) (Li et al., 2020) using GATK HaplotypeCaller (Van der Auwera et al., 2013). The RNA-seq data were obtained from the CPGEA. The raw count matrix was used by DESeq2 (Love et al., 2014) to quantify gene expression level as normalized counts. Transcripts with an adjusted *P* < 0.05 were considered differentially expressed. The AR ChIP-seq data of normal prostate epithelial and prostate cancer cells with two replicates were queried in the cistromeDB website^8^ (Zheng et al., 2019). Data passing all quality controls were selected to be visualized in the WashU epigenome browser (Zhou et al., 2011).

### Data Availability

Epigenomic data generated in this study can be visualized in the WashU Epigenome Browser.^9^ Sequencing data in FastQ format are available at the Genome Sequence Archive (GSA) for Human at the BIG Data Center,^10^ Beijing Institute of Genomics (accession number PRJCA004460). The 18-state and 15-state epigenomic maps generated using ChromHMM can be downloaded from the BIG Data Center.^11^

### Bioethics

The authors state that they obtained the approval from appropriate institutional review board and have followed the principles outlined in the Declaration of Helsinki for all human experimental research. In addition, for investigations involving human subjects, informed consent was obtained from the participants.

## Results

### Reference Map of the Normal Prostate Epigenome

The first step in the construction of a high-resolution epigenome reference is to collect high-quality data. Qualified urological pathologists curated and selected five normal adult prostate tissues (Supplementary Table 1). Chromatin immunoprecipitation sequencing (ChIP-seq) of six histone modification marks (H3K4me3, H3K4me1, H3K27ac, H3K36me3, H3K27me3, and H3K9me3), whole-genome bisulfite sequencing (WGBS), total mRNA-seq, and assay for transpose accessible chromatin using sequencing (ATAC-seq) were performed on these normal prostate specimens. The ChIP-seq datasets of H3K27ac from public resources were also integrated into our study to better define active enhancers and promoters. In total, we generated 23whole-genome datasets, including 17 ChIP-seq, 2 ATAC-seq, 3 WGBS, and 3 RNA-seq datasets (Supplementary Figure 1A). Each experiment had at least two highly correlated biological replicates, illustrated in the correlation heatmap (Supplementary Figure 1B). For ChIP-seq quality assurance, we calculated the number of usable fragments, the fraction of reads in peaks (FRiP), percentage of reads marked as duplicates, percentage of reads within blacklist regions, and relative cross-coverage scores (Supplementary Table 1). As expected, the activation-associated signals (H3K4me1, H3K4me3, H3K27ac, and H3K36me3) were characterized as having a low correlation with the repression-associated marks (H3K9me3 and H3K27me3) (Supplementary Figure 1B; Xie et al., 2013; Matsumura et al., 2015; Zhuo et al., 2020). We also used ChIP followed by quantitative polymerase chain reaction (PCR) (ChIP-qPCR) to validate some of the target regions, further confirming the high quality of the ChIP-seq data (Supplementary Figure 1C and Supplementary Table 4). For WGBS, we generated more than 9 billion bases per sample, covering 91.2% of CpGs in the whole genome with an average of 22 × coverage (Supplementary Table 1). The majority (mean 80.1%) of CpGs was methylated (gene bodies, intergenic regions, and repeats). In contrast, a small fraction of CpGs was intermediately methylated or unmethylated (CpG islands and promoters) (Supplementary Figures 2A,B), reflecting the bimodal distribution of CpG methylation levels in normal somatic cells (Supplementary Figure 2C). RNA-seq data in this study detected a total of 72% (13,828 out of 19,327) protein-coding genes expressed (TPM > 1) in the normal prostate tissue, and captured over 89% (13,368 out of 14,928) of genes detected by HPA (TPM > 1) in prostate tissues. The normalized signals of all ChIP-seq, and ATAC-seq in the gene body and the neighboring regions showed high reproducibility between replicates (Supplementary Figure 2E).

**FIGURE 1:**
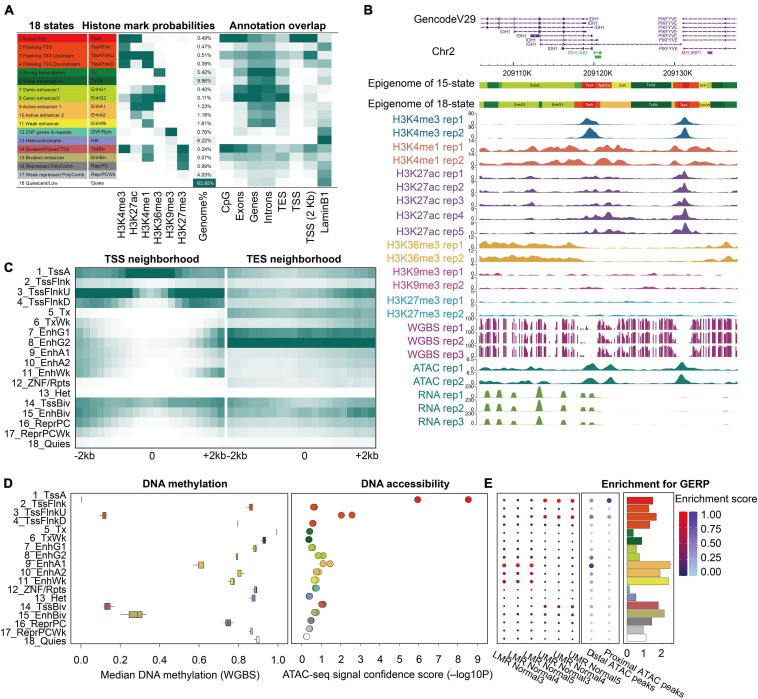
Epigenomic 18-state map of the prostate epigenome based on six histone modification marks. **(A)** Epigenomic 18 states definitions, histone mark probabilities, average genome coverage, and genomic annotation enrichments. The 18-state joint model from the Roadmap was used to generate 18 states of the prostate whole genome with the same colors and mnemonics. Different annotations of hg19 were used for the enrichment analysis. **(B)** An example region of all datasets in this study, which shows the prostate 18/15-state epigenome, six histone marks, WGBS, ATAC-seq, and RNA-seq data using the WashU Epigenome Browser. Normalized intensity of the ChIP-seq, ATAC-seq, and RNA-seq signals is shown. The values on the *y*-axis for WGBS indicate the methylation level of each CpG site. **(C)** Enrichment of the18-state epigenome in the 4-kb neighboring regions of the transcription start site (TSS) and end site (TES). **(D)** Median DNA methylation level and ATAC-seq signal confidence -log10 (*p*-value) were calculated per state. **(E)** Enrichment of lowly methylated regions, unmethylated regions (left), distal ATAC peaks, proximal ATAC peaks (middle), and GERP evolutionarily conserved non-exonic nucleotides (right).

To integrate our histone modification datasets, we first generated a stable 18-state model of the prostate epigenome using ChromHMM, following the guidelines of the Roadmap Project (Figures 1A,B). The 200-bp resolution epigenomic map of the prostate consisted of transcription signatures (1-Active TSS, 2-Flanking TSS, 3-Flanking TSS upstream, 4-Flanking TSS downstream, 5-Strong transcription, and 6-Weak transcription), enhancer signatures (7-Genic enhancer 1, 8-Genic enhancer 2, 9-Active enhancer 1, 10-Active enhancer 2, and 11-Weak enhancer), ZNF signature (12-ZNF genes and repeats), and repression signatures (13-Heterochromatin, 14-Bivalent/poised TSS, 15-Bivalent enhancer, 16-Repressed PolyComb, 17-Weak repressed PolyComb, and 18-Quiescent/Low), providing a functional annotation of the prostate genome. Simultaneously, a 15-state model of the prostate epigenome was generated with the same ChIP-seq datasets, excluding H3K27ac (Supplementary Figure 3). The biological significance of each state has been described in detail by Roadmap Epigenomics and follow-up studies (Matsumura et al., 2015; Roadmap Epigenomics Consortium et al., 2015; Pomerantz et al., 2020). We found that enhancers and promoters accounted for 5.3% (18-state) and 6.5% (15-state) of the prostate genome, respectively, and more than half of the genome was covered by the quiescent state, resembling other normal human tissues (Roadmap Epigenomics Consortium et al., 2015). To evaluate the relationship between chromatin states and genomic features, we computed the overlap and neighborhood enrichment of each state relative to specific genomic annotations (Figures 1A,C and Supplementary Figures 3A,B). We also evaluated the relationship between individual chromatin states and DNA methylation levels, as well as chromatin accessibility. Globally, the extent of activity of the regions negatively correlated with DNA methylation and positively correlated with DNA accessibility (Figure 1D and Supplementary Figure 3C). Additionally, we identified 13,565 UMRs and 65,800 LMRs using three WGBS datasets (Supplementary Table 5). We found that the enhancers were mainly enriched in the LMRs and distal ATAC peaks (Supplementary Table 2). Promoters were enriched primarily in the UMRs and proximal ATAC peaks (Figure 1E and Supplementary Figure 3D). These results underscored the chromatin signature differences between the enhancer and promoter states, which were defined by histone modifications. In addition, the enhancer states of the prostate were enriched for evolutionarily conserved non-exonic elements (Figure 1E and Supplementary Figure 3D). We found that some chromatin states showed distinct activities although they shared the same DNA accessibility patterns, such as TxFlnk, Enh, ZNF/Rpts, TssBiv, and BivFlnk. Moreover, the bivalent enhancer states (EnhBiv and BivFlnk) showed lower DNA methylation than the active enhancer states (Enh and EnhA), the biological significance of which requires future investigation (Song et al., 2019). Overall, these results demonstrate the complex relationship between DNA methylation, chromatin accessibility, and histone modifications in the prostate tissue. Studying DNA methylation or chromatin accessibility alone may have specific limitations, supporting the need for constructing a comprehensive prostate reference epigenome.

### Significant Correlation Between Multiple Epigenetic Modifications and the Prostate Transcriptome

**FIGURE 2:**
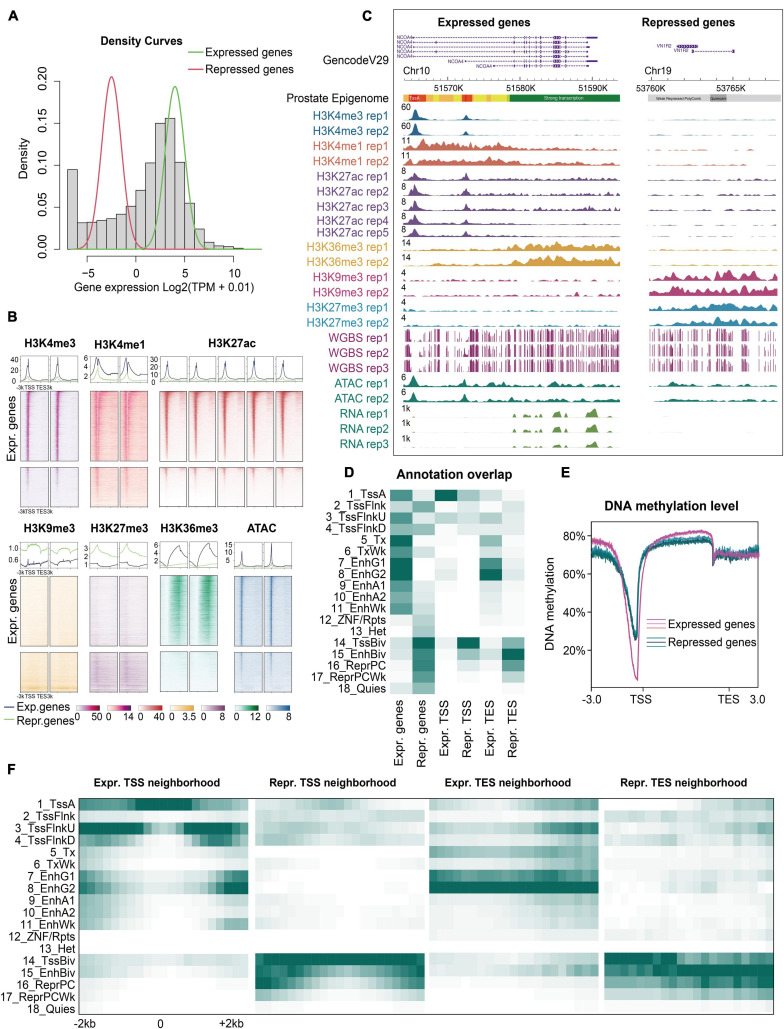
Epigenomic characteristics of expressed and repressed genes. **(A)** Density histogram. All genes were divided into two clusters: Expressed (green line) and repressed genes (red line) using a Gaussian mixture model based on the mean log2 (TPM + 0.01) value of each gene in the prostate. **(B)** The normalized signals of all ChIP-seq and ATAC-seq datasets were calculated for the expressed and repressed genes, respectively. All rows of heatmaps (top: Expressed genes, bottom: Repressed genes) are in the same descending order according to the gene expression levels. **(C)** Snapshot of an example showing the dramatically distinct epigenomic landscapes of the expressed and repressed genes using WashU Epigenome Browser. Normalized intensity of the ChIP-seq, ATAC-seq, and RNA-seq signals is shown. The values on the *y*-axis for WGBS show the methylation level of each CpG site. **(D)** Enrichment of the expressed and repressed genes, at their transcription start site (TSS) and end site (TES). **(E)** Expressed and repressed genes showing different DNA methylation signatures in three replicates. **(F)** The neighborhood of TSS and TES enrichments for the expressed and repressed genes, respectively (± 2 kb).

Having established the prostate epigenome map, we further explored the correlation between the epigenome and gene expression. Using a Gaussian mixture model (Lee, 2005), we categorized all genes into expressed and repressed genes based on RNA-seq data (Figure 2A and Supplementary Table 6). We evaluated the epigenomic patterns as a function of expression levels (Figure 2B). We found that the epigenetic signatures of the expressed and repressed genes were significantly different (Figure 2C). First, almost all the expressed genes were enriched with the active states in their bodies and regulatory regions (1_TssA, 5_Tx, 7/8_EnhG) (Figures 2D,F and Supplementary Figures 4A,B). Second, the promoters, enhancers, and gene bodies of the expressed genes showed high signals for activation-associated histone marks (H3K4me3, H3K36me3, H3K4me1, and H3K27ac), but low signals for repression-associated histone marks (H3K9me3 and H3K27me3) (Figure 2B). Third, the expressed genes displayed lower methylation levels in promoters and higher levels in gene bodies than the repressed genes (Figure 2E). In contrast, the repressed genes had unique histone marks. Two repressive signals (H3K27me3 and H3K9me3) were found with distinct distributions around the repressed genes, indicating different silencing mechanisms. For example, a small fraction of repressed genes showed high levels of H3K9me3, but low levels of H3K27me3 (Figure 2B). H3K9me3 is considered a permanent repression marker associated with heterochromatin, whereas H3K27me3 is considered a temporary repressive marker associated with PolyComb binding and CpG-rich regions (Wang et al., 2018; Jadhav et al., 2020). Therefore, the map of the prostate epigenome enables a more precise and comprehensive investigation of gene regulation in the prostate.

### Epigenome Comparison and Prostate-Specific Enhancer Modules

**FIGURE 3:**
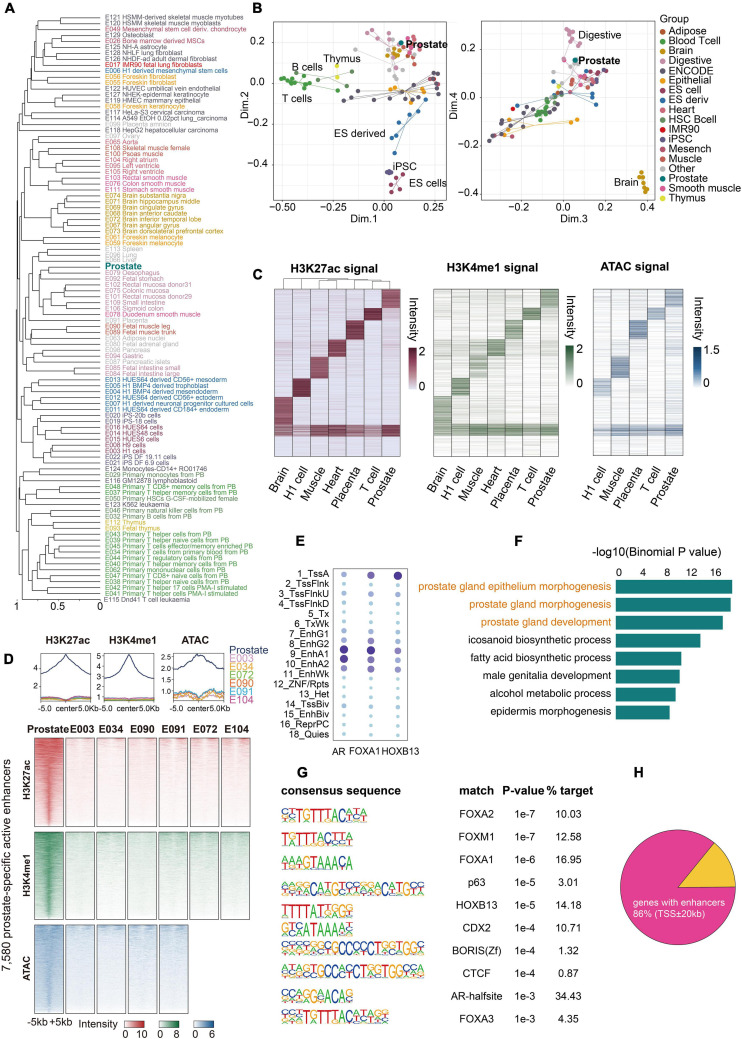
Comparison of the prostate and other epigenomes. **(A)** Hierarchical clustering of prostate and other epigenomes using all active enhancers. The active enhancer states (EnhA1 and EnhA2) of 99 epigenomes were divided into non-overlapping 200-bp bins, which were then assigned 0 (no enhancers) or 1 (enhancers). The huge matrix was used to perform hierarchical clustering. **(B)** Multidimensional scaling (MDS) plot of all 99 epigenomes based on the same matrix used for hierarchical clustering. **(C)** Clustering analysis identified tissue-specific active enhancers across the prostate and six other tissues or cell types. All active enhancers were merged into a union set of regions. Values in the heatmap were normalized RPKM (reads per kilobase million) values of H3K27ac calculated from the merged regions in each sample. Normalized H3K4me1 RPKM values for seven samples and ATAC-seq RPKM values for five samples in the corresponding enhancer clusters are shown. **(D)** H3K27ac, H3K4me1, and ATAC-seq signals of 7,580 prostate-specific active enhancers over 10-kb regions centered on the prostate demonstrate strong signals, whereas other tissues show weak signals. **(E)** Enrichments for FOXA1, AR, and HOXB13 peaks of the 18-state epigenome. **(F)** Gene ontology (GO) terms associated with the top 1,000 prostate-specific active enhancer regions using the GREAT tool for this analysis. **(G)** Enriched known motifs in the prostate-specific active enhancer regions detected by HOMER2. **(H)** A fraction of 103 genes with active prostate enhancers.

Epigenetic mechanisms are instrumental in maintaining cell identity and tissue diversity. A critical component is the enhancer module that orchestrates tissue specificity (Alvarez-Errico et al., 2015; Lee et al., 2017). We extracted all active enhancer states (EnhA1 and EnhA2) of the prostate and compared them with 98 Roadmap epigenomes of diverse tissues and cell types. Similar lineages, such as pluripotent stem cells, immune-associated cells, and brain-derived tissues, formed distinct clusters in the hierarchical clustering analysis (Figure 3A). We found that the prostate tissue clustered most closely with tissues from the digestive system. This clustering reflects that these tissues are derived from secretory organs comprising secretory epithelial and smooth muscle cells and have similar stromal components (Dedhia et al., 2016; Ikegami et al., 2020). Multidimensional scaling (MDS) analysis distinctly separated the prostate from immune cells, pluripotent stem cells, and brain tissues (Figure 3B). These results highlight that active enhancer states are a signature of specific cell types and tissue identity. Six high-quality epigenomes (E003, E034, E072, E090, E091, and E104), as representatives of distinct lineages, were selected for comparison with the prostate epigenome, and only 18.9% of active enhancers were shared across all seven tissues (Figure 3C). Moreover, we identified 7,580 prostate-specific active enhancers (see section “Materials and Methods”), which were confirmed by examining the signals of H3K27ac, H3K4me1, and ATAC (Figure 3D). Gene Ontology (GO) analysis revealed enhancer-target gene functions that were enriched in prostate gland development (Figure 3F). To construct the prostate-specific regulatory network, we defined 103 prostate-specific genes based on our RNA-seq data and GTEx project RNA-seq data (Supplementary Figure 5 and Supplementary Table 3). Of these, 89 (86%) genes had at least one active enhancer of the prostate (± 20 kb of TSS), and 77 (74.8%) genes had at least one prostate-specific active enhancer (± 20 kb of TSS) (Figure 3H and Supplementary Figures 5, 6), indicating that the active enhancers of the prostate were closely related to its identity. To identify putative master transcription factors in the prostate tissue, we performed motif enrichment analysis of prostate-specific active enhancers and found that most of them were known prostate-related master transcription factors (Figure 3G), such as FOXA1, HOXB13, and AR (Edwards et al., 2005; Hankey et al., 2020). To further study the interaction between master transcription factors and the prostate epigenome, we calculated the enrichment of FOXA1, HOXB13, and AR (ChIP-seq datasets from GSE70079 and GSE130408) in each state of the prostate epigenome. FOXA1 and AR binding was mainly enriched in enhancers, especially in active enhancers, whereas HOXB13 binding was primarily located in the promoter regions (Figure 3E and Supplementary Figures 7, 8). The global landscape of interactions among these master transcription factors and the prostate epigenome may provide valuable information for future research.

### Predicting Regulatory Functions of Disease-Associated Variants

**FIGURE 4:**
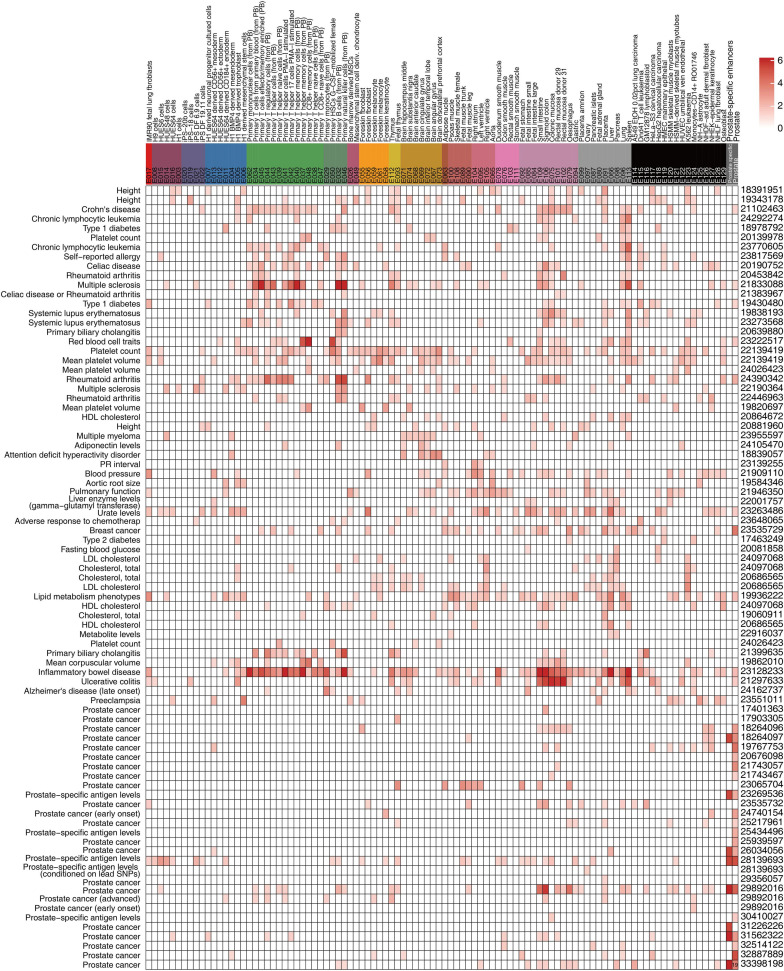
Epigenomic annotation of disease-associated variants. Enhancer states (states 8 and 9) of 18-state and prostate-specific enhancer enrichment (*p*-value < 0.05) for trait-associated genetic variants. The SNP number overlapped with the data of the prostate cancer study, and the enhancers are shown in the box. The findings of representative studies were consistent with those of the Roadmap Project.

**FIGURE 5:**
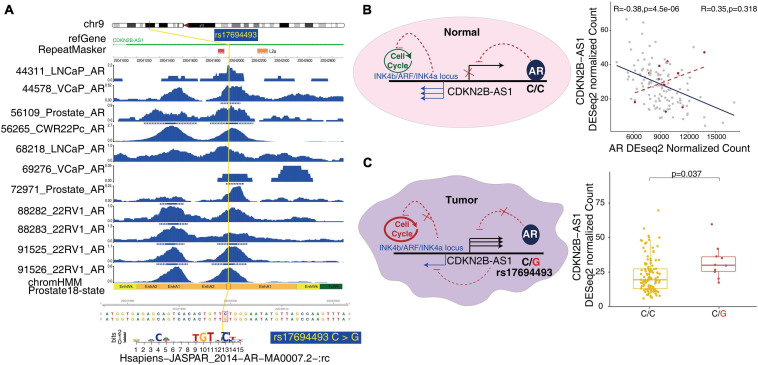
Potential mechanism of GWAS effects on tumor predisposition. **(A)** A representative GWAS locus associated with prostate cancer trait affects AR-binding motif predicted by motifbreakR in the enhancer regions. The high-quality AR ChIP-seq data from two independent studies of normal prostate epithelium and prostate cancer cell lines are shown around the representative GWAS locus. **(B)** rs17694493 is located in the intron of CDKN2B-AS1. AR binds to the SNP and inhibits CDKN2B-AS1. The Pearson correlation of CDKN2B-AS1 expression with AR in the normal prostate is demonstrated in the right panel. The groups harboring the SNP are indicated with a red dot. The anti-correlation was abolished. In the INK4b/ARF/INK4a locus, the effector genes of CDKN2B-AS1 are transcribed to control the cell cycle in the normal prostate tissue. **(C)** In tumor samples, SNP rs17694493 abolished AR binding and downstream CDKN2B-AS1 inhibition, supported by allelic expression in CPGEA prostate cancer cohorts (right panel). The upregulated expression of CDKN2B-AS1 affected INK4b/ARF/INK4a, gate guard genes of cell cycle.

To better understand the molecular mechanism underlying prostate-associated disease phenotype, we integrated the large epigenome references with trait-associated genetic variants. We obtained GWAS data for multiple diseases and traits from the University of California Santa Cruz (UCSC) Table Browser. Consistent with the results of the Roadmap Project, we confirmed that the prostate cancer-associated genetic variants were enriched in prostate-associated enhancer states (states 9 and 10 of the 18 states) (Figure 4). In the GWAS study (Conti et al., 2021), 19 out of the 186 SNPs were located in prostate enhancers. Furthermore, a substantial number of transcription factor-binding sites were created or destroyed by GWAS SNPs, including the binding sites of the androgen receptor (AR) (Supplementary Table 7). Of the 19 SNPs within the prostate enhancer regions, rs17321482 and rs17694493 were predicted to disrupt the binding of AR (Coetzee et al., 2015; Figure 5A and Supplementary Table 7). SNP rs17321482 was located in the intron of ARHGAP6 on the X chromosome. SNP rs17694493 was located on 9p21, in the intron of CDKN2B-AS1, which is a putative oncogene that encodes a long non-coding RNA, ANRIL (Walsh et al., 2014). Previous studies have predicted that the risk allele rs17694493 disrupts two transcription factor-binding motifs (STAT1 and RUNX1), which regulate the expression of the CDKN2B-CDKN2A gene cluster (Al Olama et al., 2014). However, we observed significant AR ChIP-seq signals in normal prostate epithelial and multiple prostate cancer cell lines, suggesting that the SNP overlaps a bona fide AR-binding site, and the risk allele potentially negatively influences AR binding (Figure 5A). Interestingly, when we examined data from a previously published prostate cancer cohort (Li et al., 2020), we found that AR expression negatively correlated with the expression of CDKN2B-AS1 in the normal prostate, and this correlation was completely dependent on the reference allele, but not the risk allele. This pattern was consistent with a model in which AR binding represses CDKN2B-AS1, and the disruption of the AR-binding site decouples CDKN2B-AS1 from AR control (Figure 5B). Consistent with this model, tumor samples with the risk allele rs17694493 (C > G) exhibited higher CDKN2B-AS1 expression (Figure 5C). Thus, rs17694493 might play a causal role in predisposing cancer risk.

## Discussion

International epigenomics consortia, such as the Encyclopedia of DNA Elements (ENCODE) Project (Maher, 2012), Roadmap Epigenomics Program (Roadmap Epigenomics Consortium et al., 2015), International Human Epigenome Consortium (IHEC) (Bujold et al., 2016), and Functional ANnoTation Of the Mammalian Genome 5 (FANTOM5) Consortium (Noguchi et al., 2017), have devoted great efforts to generate, analyze, and interpret epigenomics data to help understand gene regulation in development and disease. In this study, we focused on the prostate, whose complete epigenome map is lacking. Even in the most recent EpiMap (for epigenome integration across multiple projects), a compendium comprising epigenomic maps across 800 samples, a high-quality prostate epigenome is still missing (Boix et al., 2021). A complete epigenomic map of the normal prostate will likely make a significant contribution to the literature and advance research efforts on prostate cancer, the second most common cancer in men worldwide (Stelloo et al., 2018; Arap et al., 2020; Zhao et al., 2020). By integrating comprehensive histone modification ChIP-seq, WGBS, RNA-seq, and ATAC-seq data, we filled this gap, enabling comprehensive annotation of regulatory elements in normal prostate at a high resolution.

Here, we present an 18-state epigenome map of the normal prostate and analyzed the patterns of DNA methylation and chromatin accessibility in each state. In most cases, the analyses confirmed the general knowledge about DNA methylation and chromatin accessibility levels in relation to epigenome states; however, some interesting exceptions were observed. For instance, bivalent enhancers showed lower DNA methylation levels than active enhancers but had similar chromatin accessibility. Previous studies suggested that DNA methylation can be antagonistic to H3K27me3 in enhancer regions (Inoue et al., 2017; Chen et al., 2019). Therefore, chromatin accessibility and DNA methylation work in conjunction with other histone modifications rather than independently or redundantly, to control gene regulation (Gal-Yam et al., 2008; Bogdanovic et al., 2011; King et al., 2016). The complex relationship between chromatin accessibility, DNA methylation, and chromatin states in the prostate remains to be elucidated.

We also found that enhancer and promoter states accounted for 5.3% of the prostate genome, and they exhibited increased evolutionary conservation, underscoring the biological significance of these regions. We defined 5,625 prostate-specific active enhancers, demonstrated their potential to distinguish prostate tissue identity, and investigated their association with prostate-specific gene expression.

Furthermore, we examined the master transcription factors in the prostate, including FOXA1, AR, and HOXB13. Our previous study confirmed that *FOXA1* was frequently mutated in prostate cancer in an Asian cohort (Li et al., 2020). Recent studies have also found that *FOXA1* mutations affect the phenotype of prostate cancer and interfere with the differentiation of normal prostate epithelium (Adams et al., 2019; Parolia et al., 2019). By defining a global landscape of interactions between FOXA1 and prostate-specific regulatory elements, especially active enhancers, we provide a useful resource for future research. We also found the motifs of CTCF and BORIS (Zf) in prostate-specific enhancers, suggesting the existence of prostate-specific chromatin interactions (Rowley and Corces, 2018; Debruyne et al., 2019).

Finally, to illustrate the utility of our prostate epigenome map, we used this map to annotate genetic variants that are associated with disease traits. The current NHGRI GWAS catalog has collected over four thousand GWAS studies (Welter et al., 2014). However, functionalizing trait-associated genetic variants has been a major challenge. The majority of GWAS SNPs reside in non-coding regions, which are potentially regulatory elements. Integrating large epigenomic roadmaps holds promise to provide a principled approach to elucidate the functional consequences of GWAS SNPs. In our study, we used the prostate-specific enhancer to link a novel GWAS SNP with upstream AR binding and downstream disturbance of cell cycle regulation. We suggest that it is a promising paradigm to integrate the epigenome reference, public data, and large tumor consortium to interpret and identify possible causal variants.

In summary, our normal prostate epigenome map complements the current human reference epigenome and fills an important gap in the field. This is valuable for a better understanding of gene regulation, development, and tumorigenesis of the prostate. Further studies will be required to investigate the complex relationship between chromatin accessibility, DNA methylation, histone modifications, and chromatin states in the prostate, and to validate mechanistic predictions on the functional consequences of genetic variations.

## Data Availability Statement

The datasets presented in this study can be found in online repositories. The names of the repository/repositories and accession number(s) can be found in the article/Supplementary Material.

## Ethics Statement

The studies involving human participants were reviewed and approved by the ethical committee approval was secured from the Changhai Hospital (CHEC2019-012). The patients/participants provided their written informed consent to participate in this study.

## Author Contributions

JL, JY, and BL conceived the overall project and directed its execution in their labs. MQ, LZ, and YW were mainly responsible for collecting all specimens. JS, WZ, and TW performed the experiments in Bing’s lab. TW and ZW performed integrative computational analyses of the epigenomic data. All authors read and approved the final manuscript.

## Conflict of Interest

The authors declare that the research was conducted in the absence of any commercial or financial relationships that could be construed as a potential conflict of interest.

## Publisher’s Note

All claims expressed in this article are solely those of the authors and do not necessarily represent those of their affiliated organizations, or those of the publisher, the editors and the reviewers. Any product that may be evaluated in this article, or claim that may be made by its manufacturer, is not guaranteed or endorsed by the publisher.

## Footnotes

1
http://www.bioinformatics.babraham.ac.uk/projects/fastqc/

2
http://broadinstitute.github.io/picard

3
https://www.ncbi.nlm.nih.gov/books/NBK158900/

4
http://mendel.stanford.edu/SidowLab/downloads/gerp/

5
https://egg2.wustl.edu/roadmap/data/byFileType/chromhmmSegmentations/ChmmModels/core_K27ac/jointModel/final/

6
https://www.proteinatlas.org/

7
https://gtexportal.org/home/datasets

8
http://www.cistrome.org/db/#/

9
https://epigenomegateway.wustl.edu/browser/?genome=hg19&hub=https://epigenome.w0075stl.edu/normal_prostate_epigenome/hub

10
http://bigd.big.ac.cn/gsa-human/

11
https://bigd.big.ac.cn/omix/release/OMIX237

## References

[B1] Adams E. J. Karthaus W. R. Hoover E. Liu D. Gruet A. Zhang Z. (2019). FOXA1 mutations alter pioneering activity, differentiation and prostate cancer phenotypes. *Nature* 571 408–412. 10.1038/s41586-019-1318-9 31243370 PMC6661172

[B2] Al Olama A. A. Kote-Jarai Z. Berndt S. I. Conti D. V. Schumacher F. Han Y. (2014). A meta-analysis of 87,040 individuals identifies 23 new susceptibility loci for prostate cancer. *Nat. Genet.* 46 1103–1109. 10.1038/ng.3094 25217961 PMC4383163

[B3] Alvarez-Errico D. Vento-Tormo R. Sieweke M. Ballestar E. (2015). Epigenetic control of myeloid cell differentiation, identity and function. *Nat. Rev. Immunol.* 15 7–17. 10.1038/nri3777 25534619

[B4] Arap W. Pasqualini R. Costello J. F. (2020). Prostate cancer progression and the epigenome. *N. Engl. J. Med.* 383 2287–2290. 10.1056/NEJMcibr2030475 33264551

[B5] Bogdanovic O. Long S. W. van Heeringen S. J. Brinkman A. B. Gomez-Skarmeta J. L. Stunnenberg H. G. (2011). Temporal uncoupling of the DNA methylome and transcriptional repression during embryogenesis. *Genome Res.* 21 1313–1327. 10.1101/gr.114843.110 21636662 PMC3149498

[B6] Boix C. A. James B. T. Park Y. P. Meuleman W. Kellis M. (2021). Regulatory genomic circuitry of human disease loci by integrative epigenomics. *Nature* 590:3145. 10.1038/s41586-020-03145-z 33536621 PMC7875769

[B7] Bolger A. M. Lohse M. Usadel B. (2014). Trimmomatic: a flexible trimmer for Illumina sequence data. *Bioinformatics* 30 2114–2120. 10.1093/bioinformatics/btu170 24695404 PMC4103590

[B8] Bujold D. Morais D. A. L. Gauthier C. Cote C. Caron M. Kwan T. (2016). The international human epigenome consortium data portal. *Cell Syst.* 3 496–499. 10.1016/j.cels.2016.10.019 27863956

[B9] Burger L. Gaidatzis D. Schubeler D. Stadler M. B. (2013). Identification of active regulatory regions from DNA methylation data. *Nucleic Acids Res.* 41:e155. 10.1093/nar/gkt599 23828043 PMC3763559

[B10] Carroll T. S. Liang Z. Salama R. Stark R. de Santiago I. (2014). Impact of artifact removal on ChIP quality metrics in ChIP-seq and ChIP-exo data. *Front. Genet.* 5:75. 10.3389/fgene.2014.00075 24782889 PMC3989762

[B11] Cavalli G. Heard E. (2019). Advances in epigenetics link genetics to the environment and disease. *Nature* 571 489–499. 10.1038/s41586-019-1411-0 31341302

[B12] Chen L. Ge B. Casale F. P. Vasquez L. Kwan T. Garrido-Martin D. (2016). Genetic drivers of epigenetic and transcriptional variation in human immune cells. *Cell* 167:e1324. 10.1016/j.cell.2016.10.026 27863251 PMC5119954

[B13] Chen Z. Yin Q. Inoue A. Zhang C. Zhang Y. (2019). Allelic H3K27me3 to allelic DNA methylation switch maintains noncanonical imprinting in extraembryonic cells. *Sci. Adv.* 5:eaay7246. 10.1126/sciadv.aay7246 32064321 PMC6989337

[B14] Coetzee S. G. Coetzee G. A. Hazelett D. J. (2015). motifbreakR: an R/Bioconductor package for predicting variant effects at transcription factor binding sites. *Bioinformatics* 31 3847–3849. 10.1093/bioinformatics/btv470 26272984 PMC4653394

[B15] Collins F. S. Morgan M. Patrinos A. (2003). The Human Genome Project: lessons from large-scale biology. *Science* 300 286–290. 10.1126/science.1084564 12690187

[B16] Conti D. V. Darst B. F. Moss L. C. Saunders E. J. Sheng X. Chou A. (2021). Trans-ancestry genome-wide association meta-analysis of prostate cancer identifies new susceptibility loci and informs genetic risk prediction. *Nat. Genet.* 53 65–75. 10.1038/s41588-020-00748-0 33398198 PMC8148035

[B17] Corces M. R. Trevino A. E. Hamilton E. G. Greenside P. G. Sinnott-Armstrong N. A. Vesuna S. (2017). An improved ATAC-seq protocol reduces background and enables interrogation of frozen tissues. *Nat. Methods* 14 959–962. 10.1038/nmeth.4396 28846090 PMC5623106

[B18] Cornford P. van den Bergh R. C. N. Briers E. Van den Broeck T. Cumberbatch M. G. De Santis M. (2021). EAU-EANM-ESTRO-ESUR-SIOG guidelines on prostate cancer. part ii-2020 update: treatment of relapsing and metastatic prostate cancer. *Eur. Urol.* 79 263–282. 10.1016/j.eururo.2020.09.046 33039206

[B19] Debruyne D. N. Dries R. Sengupta S. Seruggia D. Gao Y. Sharma B. (2019). BORIS promotes chromatin regulatory interactions in treatment-resistant cancer cells. *Nature* 572 676–680. 10.1038/s41586-019-1472-0 31391581 PMC7010522

[B20] Dedhia P. H. Bertaux-Skeirik N. Zavros Y. Spence J. R. (2016). Organoid models of human gastrointestinal development and disease. *Gastroenterology* 150 1098–1112. 10.1053/j.gastro.2015.12.042 26774180 PMC4842135

[B21] Dobin A. Davis C. A. Schlesinger F. Drenkow J. Zaleski C. Jha S. (2013). STAR: ultrafast universal RNA-seq aligner. *Bioinformatics* 29 15–21. 10.1093/bioinformatics/bts635 23104886 PMC3530905

[B22] Edwards S. Campbell C. Flohr P. Shipley J. Giddings I. Te-Poele R. (2005). Expression analysis onto microarrays of randomly selected cDNA clones highlights HOXB13 as a marker of human prostate cancer. *Br. J. Cancer* 92 376–381. 10.1038/sj.bjc.6602261 15583692 PMC2361840

[B23] Ernst J. Kellis M. (2012). ChromHMM: automating chromatin-state discovery and characterization. *Nat. Methods* 9 215–216. 10.1038/nmeth.1906 22373907 PMC3577932

[B24] Ernst J. Kheradpour P. Mikkelsen T. S. Shoresh N. Ward L. D. Epstein C. B. (2011). Mapping and analysis of chromatin state dynamics in nine human cell types. *Nature* 473 43–49. 10.1038/nature09906 21441907 PMC3088773

[B25] Gal-Yam E. N. Egger G. Iniguez L. Holster H. Einarsson S. Zhang X. (2008). Frequent switching of Polycomb repressive marks and DNA hypermethylation in the PC3 prostate cancer cell line. *Proc. Natl. Acad. Sci. USA* 105 12979–12984. 10.1073/pnas.0806437105 18753622 PMC2529074

[B26] Hankey W. Chen Z. Wang Q. (2020). Shaping chromatin states in prostate cancer by pioneer transcription factors. *Cancer Res.* 80 2427–2436. 10.1158/0008-5472.CAN-19-3447 32094298 PMC7299826

[B27] Harrow J. Frankish A. Gonzalez J. M. Tapanari E. Diekhans M. Kokocinski F. (2012). GENCODE: the reference human genome annotation for The ENCODE Project. *Genome Res.* 22 1760–1774. 10.1101/gr.135350.111 22955987 PMC3431492

[B28] Heinz S. Benner C. Spann N. Bertolino E. Lin Y. C. Laslo P. (2010). Simple combinations of lineage-determining transcription factors prime cis-regulatory elements required for macrophage and B cell identities. *Mol. Cell* 38 576–589. 10.1016/j.molcel.2010.05.004 20513432 PMC2898526

[B29] Ikegami K. Secchia S. Almakki O. Lieb J. D. Moskowitz I. P. (2020). Phosphorylated lamin a/c in the nuclear interior binds active enhancers associated with abnormal transcription in progeria. *Dev. Cell* 52:e611. 10.1016/j.devcel.2020.02.011 32208162 PMC7201903

[B30] Inoue A. Jiang L. Lu F. Suzuki T. Zhang Y. (2017). Maternal H3K27me3 controls DNA methylation-independent imprinting. *Nature* 547 419–424. 10.1038/nature23262 28723896 PMC9674007

[B31] Jadhav U. Manieri E. Nalapareddy K. Madha S. Chakrabarti S. Wucherpfennig K. (2020). Replicational dilution of H3K27me3 in mammalian cells and the role of poised promoters. *Mol. Cell* 78:e145. 10.1016/j.molcel.2020.01.017 32027840 PMC7376365

[B32] King A. D. Huang K. Rubbi L. Liu S. Wang C. Y. Wang Y. (2016). Reversible regulation of promoter and enhancer histone landscape by DNA methylation in mouse embryonic stem cells. *Cell Rep.* 17 289–302. 10.1016/j.celrep.2016.08.083 27681438 PMC5507178

[B33] Kovaka S. Zimin A. V. Pertea G. M. Razaghi R. Salzberg S. L. Pertea M. (2019). Transcriptome assembly from long-read RNA-seq alignments with StringTie2. *Genome Biol.* 20:278. 10.1186/s13059-019-1910-1 31842956 PMC6912988

[B34] Krueger F. Andrews S. R. (2011). Bismark: a flexible aligner and methylation caller for Bisulfite-Seq applications. *Bioinformatics* 27 1571–1572. 10.1093/bioinformatics/btr167 21493656 PMC3102221

[B35] Lee D. S. (2005). Effective gaussian mixture learning for video background subtraction. *IEEE Trans. Pattern Anal. Mach. Intell.* 27 827–832. 10.1109/TPAMI.2005.102 15875805

[B36] Lee J. E. Park Y. K. Park S. Jang Y. Waring N. Dey A. (2017). Brd4 binds to active enhancers to control cell identity gene induction in adipogenesis and myogenesis. *Nat. Commun.* 8:2217. 10.1038/s41467-017-02403-5 29263365 PMC5738375

[B37] Li H. Durbin R. (2009). Fast and accurate short read alignment with Burrows-Wheeler transform. *Bioinformatics* 25 1754–1760. 10.1093/bioinformatics/btp324 19451168 PMC2705234

[B38] Li H. Handsaker B. Wysoker A. Fennell T. Ruan J. Homer N. (2009). The sequence alignment/map format and SAMtools. *Bioinformatics* 25 2078–2079. 10.1093/bioinformatics/btp352 19505943 PMC2723002

[B39] Li J. Xu C. Lee H. J. Ren S. Zi X. Zhang Z. (2020). A genomic and epigenomic atlas of prostate cancer in Asian populations. *Nature* 580 93–99. 10.1038/s41586-020-2135-x 32238934

[B40] Love M. I. Huber W. Anders S. (2014). Moderated estimation of fold change and dispersion for RNA-seq data with DESeq2. *Genome Biol.* 15 550. 10.1186/s13059-014-0550-8 25516281 PMC4302049

[B41] Maher B. (2012). ENCODE: The human encyclopaedia. *Nature* 489 46–48. 10.1038/489046a 22962707

[B42] Matsumura Y. Nakaki R. Inagaki T. Yoshida A. Kano Y. Kimura H. (2015). H3K4/H3K9me3 bivalent chromatin domains targeted by lineage-specific DNA methylation pauses adipocyte differentiation. *Mol. Cell* 60 584–596. 10.1016/j.molcel.2015.10.025 26590716

[B43] McLean C. Y. Bristor D. Hiller M. Clarke S. L. Schaar B. T. Lowe C. B. (2010). GREAT improves functional interpretation of cis-regulatory regions. *Nat. Biotechnol.* 28 495–501. 10.1038/nbt.1630 20436461 PMC4840234

[B44] Noguchi S. Arakawa T. Fukuda S. Furuno M. Hasegawa A. Hori F. (2017). FANTOM5 CAGE profiles of human and mouse samples. *Sci. Data* 4:170112. 10.1038/sdata.2017.112 28850106 PMC5574368

[B45] Parolia A. Cieslik M. Chu S. C. Xiao L. Ouchi T. Zhang Y. (2019). Distinct structural classes of activating FOXA1 alterations in advanced prostate cancer. *Nature* 571 413–418. 10.1038/s41586-019-1347-4 31243372 PMC6661908

[B46] Pomerantz M. M. Qiu X. Zhu Y. Takeda D. Y. Pan W. Baca S. C. (2020). Prostate cancer reactivates developmental epigenomic programs during metastatic progression. *Nat. Genet.* 52 790–799. 10.1038/s41588-020-0664-8 32690948 PMC10007911

[B47] Ramirez F. Ryan D. P. Gruning B. Bhardwaj V. Kilpert F. Richter A. S. (2016). deepTools2: a next generation web server for deep-sequencing data analysis. *Nucleic Acids Res.* 44 W160–W165. 10.1093/nar/gkw257 27079975 PMC4987876

[B48] Rhie S. K. Perez A. A. Lay F. D. Schreiner S. Shi J. Polin J. (2019). A high-resolution 3D epigenomic map reveals insights into the creation of the prostate cancer transcriptome. *Nat. Commun.* 10:4154. 10.1038/s41467-019-12079-8 31515496 PMC6742760

[B49] Roadmap Epigenomics Consortium, Kundaje A. Meuleman W. Ernst J. Bilenky M. Yen A. (2015). Integrative analysis of 111 reference human epigenomes. *Nature* 518 317–330. 10.1038/nature14248 25693563 PMC4530010

[B50] Rowley M. J. Corces V. G. (2018). Organizational principles of 3D genome architecture. *Nat. Rev. Genet.* 19 789–800. 10.1038/s41576-018-0060-8 30367165 PMC6312108

[B51] Scrucca L. Fop M. Murphy T. B. Raftery A. E. (2016). mclust 5: clustering, classification and density estimation using gaussian finite mixture models. *R J* 8 289–317. 27818791 PMC5096736

[B52] Song Y. van den Berg P. R. Markoulaki S. Soldner F. Dall’Agnese A. Henninger J. E. (2019). Dynamic enhancer DNA methylation as basis for transcriptional and cellular heterogeneity of ESCs. *Mol. Cell* 75:e906. 10.1016/j.molcel.2019.06.045 31422875 PMC6731151

[B53] Stelloo S. Nevedomskaya E. Kim Y. Schuurman K. Valle-Encinas E. Lobo J. (2018). Integrative epigenetic taxonomy of primary prostate cancer. *Nat. Commun.* 9:4900. 10.1038/s41467-018-07270-2 30464211 PMC6249266

[B54] Uhlen M. Fagerberg L. Hallstrom B. M. Lindskog C. Oksvold P. Mardinoglu A. (2015). Proteomics. Tissue-based map of the human proteome. *Science* 347:1260419. 10.1126/science.1260419 25613900

[B55] Van der Auwera G. A. Carneiro M. O. Hartl C. Poplin R. Del Angel G. Levy-Moonshine A. (2013). From FastQ data to high confidence variant calls: the Genome Analysis Toolkit best practices pipeline. *Curr. Protoc. Bioinform.* 43:1033. 10.1002/0471250953.bi1110s43 25431634 PMC4243306

[B56] van der Harst P. de Windt L. J. Chambers J. C. (2017). Translational perspective on epigenetics in cardiovascular disease. *J. Am. Coll. Cardiol.* 70 590–606. 10.1016/j.jacc.2017.05.067 28750703 PMC5543329

[B57] Verze P. Cai T. Lorenzetti S. (2016). The role of the prostate in male fertility, health and disease. *Nat. Rev. Urol.* 13 379–386. 10.1038/nrurol.2016.89 27245504

[B58] Waddington C. H. (2012). The epigenotype. 1942. *Int. J. Epidemiol.* 41 10–13. 10.1093/ije/dyr184 22186258

[B59] Walsh A. L. Tuzova A. V. Bolton E. M. Lynch T. H. Perry A. S. (2014). Long noncoding RNAs and prostate carcinogenesis: the missing ‘linc’? *Trends Mol. Med.* 20 428–436. 10.1016/j.molmed.2014.03.005 24836411

[B60] Wang C. Liu X. Gao Y. Yang L. Li C. Liu W. (2018). Reprogramming of H3K9me3-dependent heterochromatin during mammalian embryo development. *Nat. Cell Biol.* 20 620–631. 10.1038/s41556-018-0093-4 29686265

[B61] Welter D. MacArthur J. Morales J. Burdett T. Hall P. Junkins H. (2014). The NHGRI GWAS Catalog, a curated resource of SNP-trait associations. *Nucleic Acids Res.* 42 D1001–D1006. 10.1093/nar/gkt1229 24316577 PMC3965119

[B62] Xie W. Schultz M. D. Lister R. Hou Z. Rajagopal N. Ray P. (2013). Epigenomic analysis of multilineage differentiation of human embryonic stem cells. *Cell* 153 1134–1148. 10.1016/j.cell.2013.04.022 23664764 PMC3786220

[B63] Yu V. W. C. Yusuf R. Z. Oki T. Wu J. Saez B. Wang X. (2016). Epigenetic memory underlies cell-autonomous heterogeneous behavior of hematopoietic stem cells. *Cell* 167:e1317. 10.1016/j.cell.2016.10.045 27863245

[B64] Zhang Y. Liu T. Meyer C. A. Eeckhoute J. Johnson D. S. Bernstein B. E. (2008). Model-based analysis of ChIP-Seq (MACS). *Genome Biol.* 9:R137. 10.1186/gb-2008-9-9-r137 18798982 PMC2592715

[B65] Zhao S. G. Chen W. S. Li H. Foye A. Zhang M. Sjostrom M. (2020). The DNA methylation landscape of advanced prostate cancer. *Nat. Genet.* 52 778–789. 10.1038/s41588-020-0648-8 32661416 PMC7454228

[B66] Zheng R. Wan C. Mei S. Qin Q. Wu Q. Sun H. (2019). Cistrome Data Browser: expanded datasets and new tools for gene regulatory analysis. *Nucleic Acids Res.* 47 D729–D735. 10.1093/nar/gky1094 30462313 PMC6324081

[B67] Zhou X. Maricque B. Xie M. Li D. Sundaram V. Martin E. A. (2011). The human epigenome browser at washington university. *Nat. Methods* 8 989–990. 10.1038/nmeth.1772 22127213 PMC3552640

[B68] Zhuo X. Du A. Y. Pehrsson E. C. Li D. Wang T. (2020). Epigenomic differences in the human and chimpanzee genomes are associated with structural variation. *Genome Res.* 2020:120. 10.1101/gr.263491.120 33303495 PMC7849402

