# Supplementary material for: Integrative Epigenome Map of the Normal Human Prostate Provides Insights Into Prostate Cancer Predisposition
Source: Front Cell Dev Biol. 2021 Aug 26;9:723676. doi: 10.3389/fcell.2021.723676 (PMC8427514; doi:10.3389/fcell.2021.723676)
Supplement: Supplementary file 8 [file Data_Sheet_1.PDF]

SUPPLEMENTARY INFORMATION

Supplementary Figure 1. Epigenetic datasets and ChIP-qPCR results.

A. Data availability for six histone marks, DNA methylation, chromatin accessibility, and RNA-seq. Columns represent individual normal prostate samples. H3K27ac of three replicates from GSE130408. B. A correlation heatmap based on all peak sets. Pearson correlation coefficients were calculated to perform the clustering of all chromatin immunoprecipitation sequencing (ChIP-seq) datasets. C. Chromatin immunoprecipitation sequencing using quantitative polymerase chain reaction (ChIP-qPCR) for six histone modifications. For each histone mark, distinct primers for the positive and negative regions were designed (Supplementary Table 5). The percentage of input was calculated as 100 × 2^ (Cpadjusted input - Cptreatment) and used to show the relative enrichment of positive regions in two replicate samples. The Cp of the input DNA was adjusted from 5% to 100% equivalent.

Supplementary Figure 2. Landscape of different epigenetic datasets.

A. Global mean methylation levels of CpGs (mCG/CG) and fraction of total CpGs with low, medium, and high methylation levels. B. Mean methylation level of CpGs in distinct genomic features or repetitive element classes. C. Density plot of genome-wide DNA methylation levels of all CpGs. D. Pie charts showing the detected genes in the prostate tissue. E. The normalized signals of all ChIP-seq, and transposase-accessible chromatin sequencing (ATAC-seq) in the gene body and its 3-kb or 30-kb (H3K9me3) upstream and downstream regions.

Supplementary Figure 3. Epigenomic 15-state map of prostate reference epigenome based on five histone modification marks.

A. Epigenomic 15 states definitions, histone mark probabilities, average genome coverage, and genomic annotation enrichments. The 15-state joint model from the Roadmap was used to generate 15 states of the prostate whole genome with the same colors and mnemonics. B. Enrichments of the 15-state epigenome in the 4-kb neighboring regions of TSS and TES. C. Median DNA methylation level and transposase-accessible chromatin sequencing (ATAC-seq) signal confidence -log10 (p-value) were calculated for each state. D. Enrichments of low-methylated, unmethylated regions (left), distal ATAC peaks, proximal ATAC peaks (middle), and GERP evolutionarily conserved non-exonic nucleotides (right).

Supplementary Figure 4. Expressed and repressed genes with similar differences in the prostate 15-state epigenome.

A. Expressed and repressed gene enrichments for a 15-state epigenome of the prostate. B. The neighboring region (± 2 kb) of the transcription start site (TSS) and end site (TES) enrichments for the expressed and repressed genes, respectively.

Supplementary Figure 5. Heatmap showing the mean log2 (TPM+0.01) values of 103 prostate-specific genes of 55 normal human tissues from GTEx. The first two columns on the left represent the normal prostate samples from GTEx and this study. The gene-related enhancer was defined as within 20 kb upstream and downstream of TSS. Prostate-specific genes were divided into three groups: 1) prostate-enriched genes with at least four-fold higher mRNA levels in the prostate compared to any other tissue; 2) group-enriched genes with at least four-fold higher average mRNA levels in a group of 2–5 tissues, including the prostate, compared to any other tissues; and 3) prostate-enhanced genes with at least four-fold higher mRNA levels in the prostate compared to the average level in all other tissues.

Supplementary Figure 6. Snapshot of prostate-specific genes with prostate-specific active enhancers using WashU Epigenome Browser.

The epigenome tracks of the prostate and six other tissue or cell types, and normalized intensity of the ChIP-seq signal for H3K27ac, AR, FOXA1, and ATAC-seq data are shown.

Supplementary Figure 7. Enrichments for FOXA1, AR, and HOXB13 peaks of the 15-state epigenome.

FOXA1 and AR binding was mainly enriched with the enhancers, whereas HOXB13 binding was primarily located in the promoter regions.

Supplementary Figure 8. HOXB13 ChIP-seq signals over 10-kb regions centered on promoter signatures (TssA) and active enhancer signatures (EnhA1 and EnhA2).

Compared with enhancer signatures, promoter signatures showed stronger signals of HOXB13 ChIP-seq data, indicating that HOXB13 binding was primarily located in the promoter regions.

Supplementary Table 1. Clinical information and sequencing quality metrics of all samples.

Supplementary Table 2. Distal ATAC peaks and proximal ATAC peaks.

Supplementary Table 3. List of 103 prostate-specific genes.

Supplementary Table 4. Primers designed for ChIP-qPCR experiment.

Supplementary Table 5. Unmethylated regions (UMRs) and lowly methylated regions (LMRs).

Supplementary Table 6. Expressed and repressed genes in the prostate.

Supplementary Table 7. GWAS traits enrichment and TF motif prediction.
