# Supplementary figures and images for: Integrative Epigenome Map of the Normal Human Prostate Provides Insights Into Prostate Cancer Predisposition
Source: Front Cell Dev Biol. 2021 Aug 26;9:723676. doi: 10.3389/fcell.2021.723676 (PMC8427514; doi:10.3389/fcell.2021.723676)

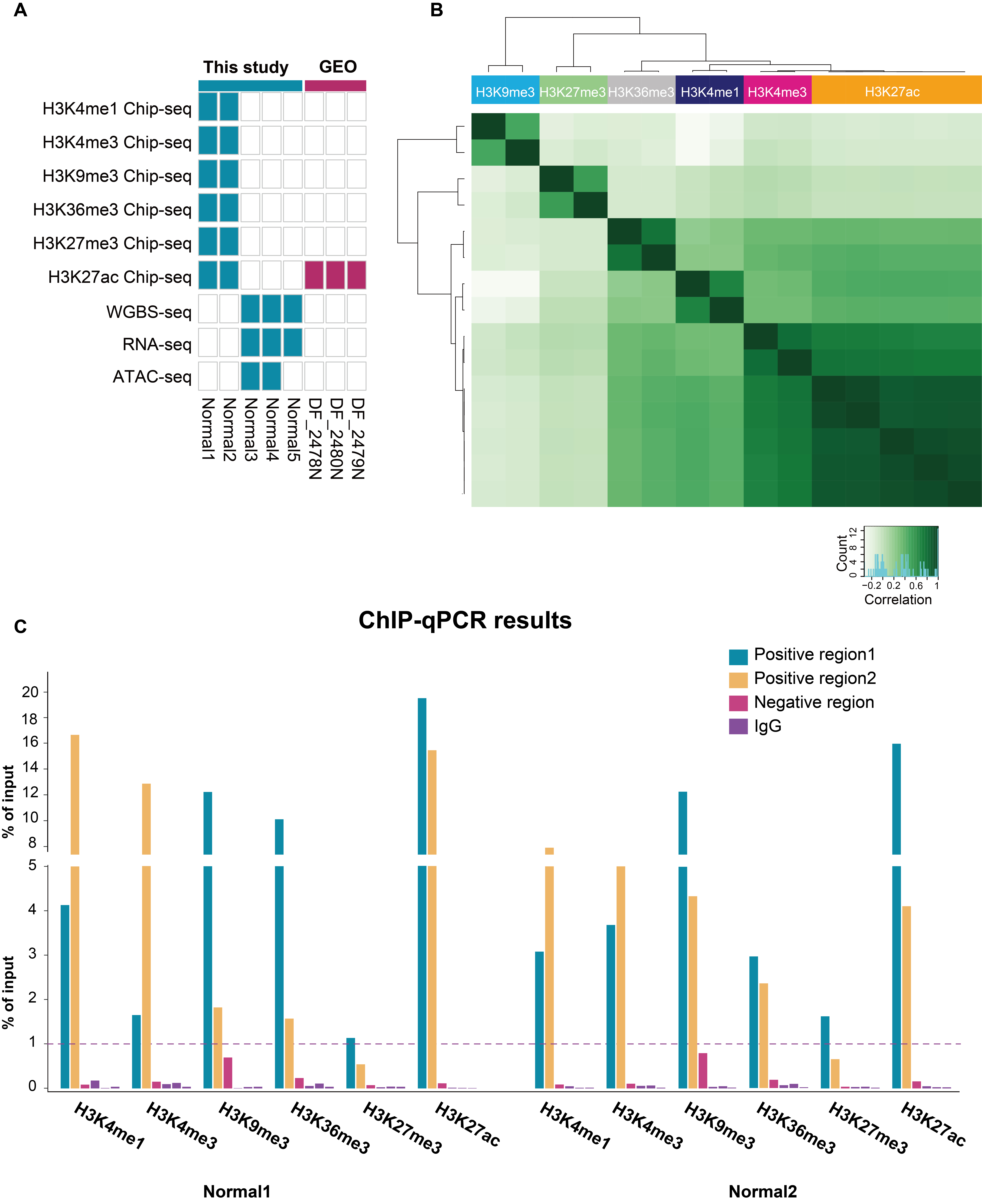

Supplement: Supplementary file 9 [file Image_1.JPEG]

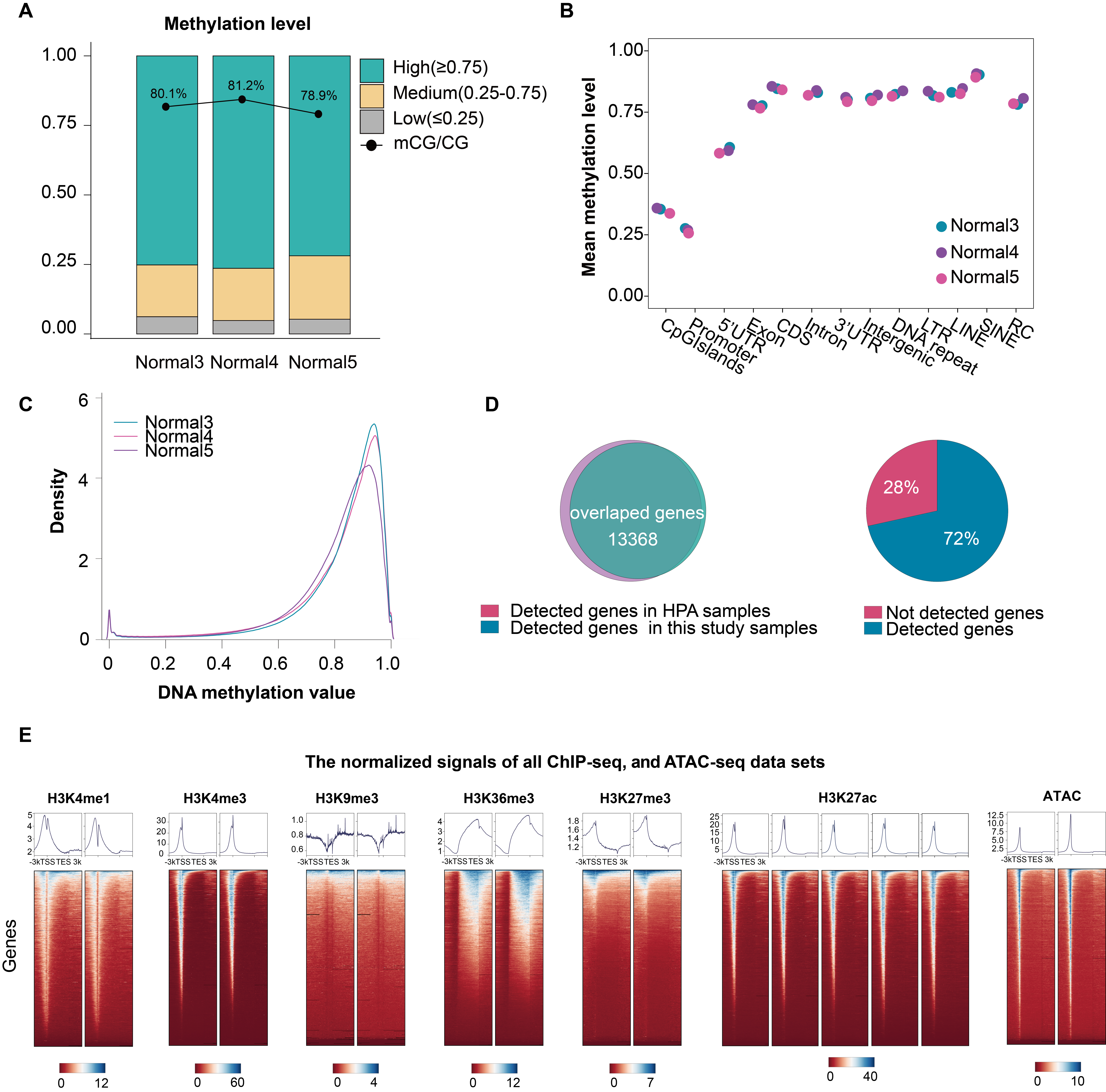

Supplement: Supplementary file 10 [file Image_2.JPEG]

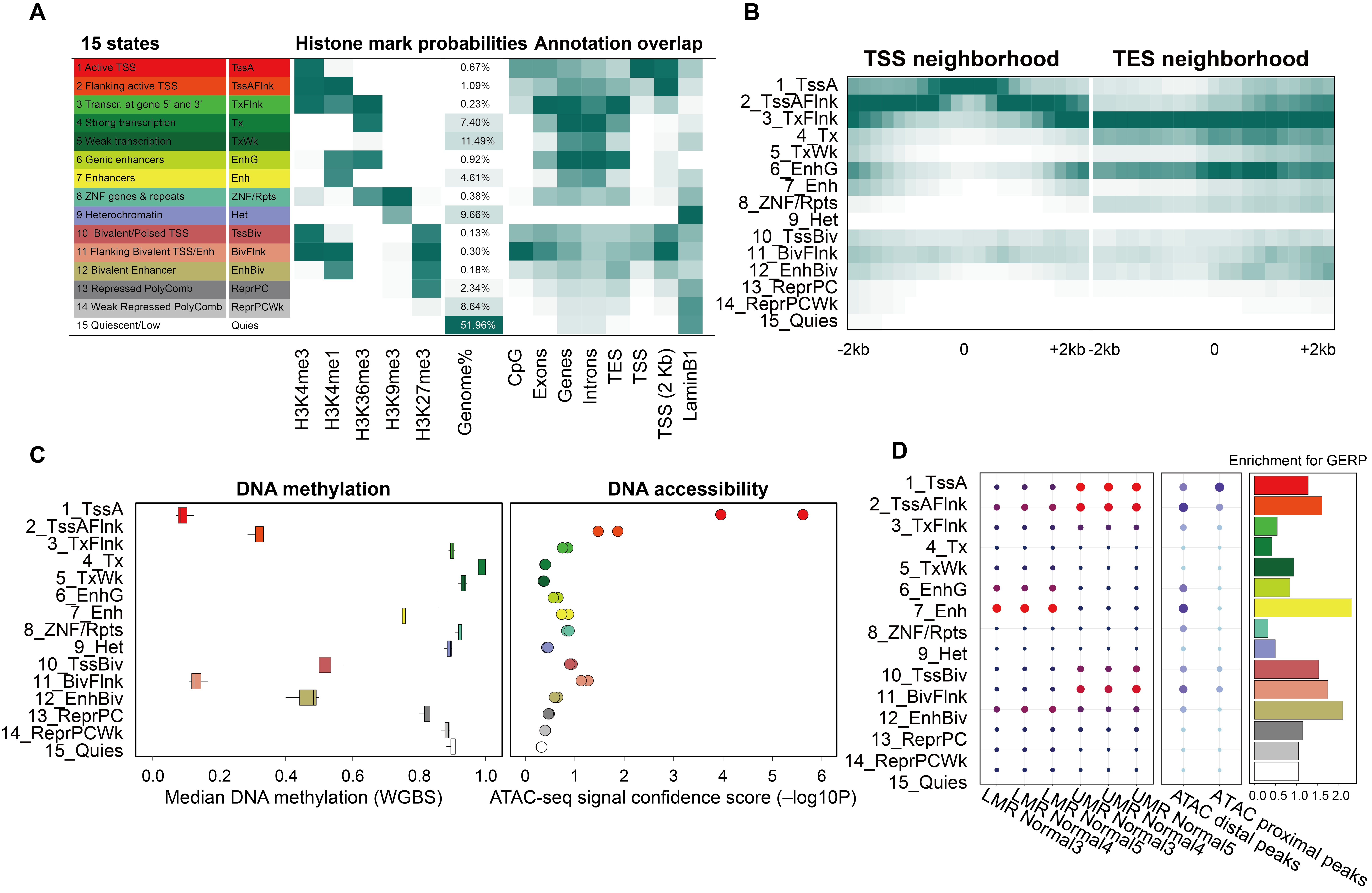

Supplement: Supplementary file 11 [file Image_3.JPEG]

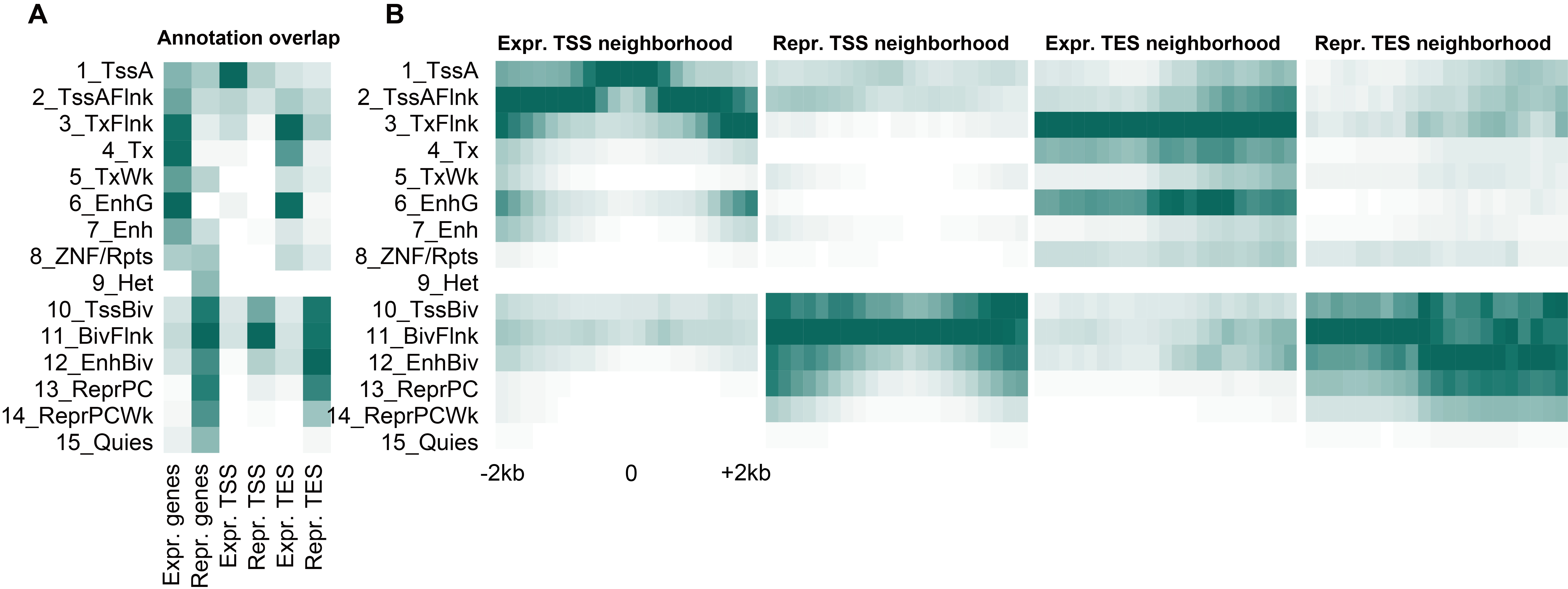

Supplement: Supplementary file 12 [file Image_4.JPEG]

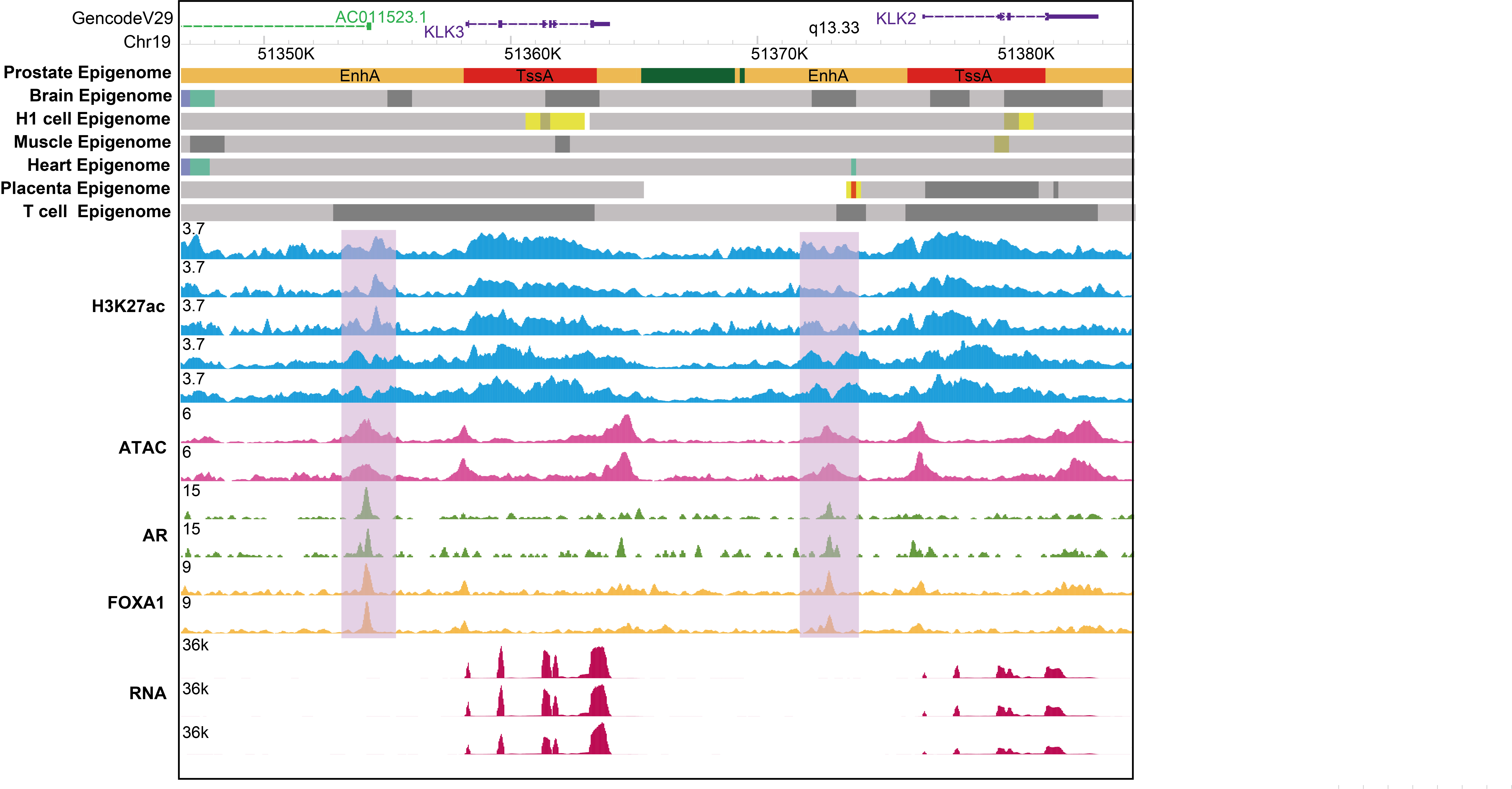

Supplement: Supplementary file 14 [file Image_6.JPEG]

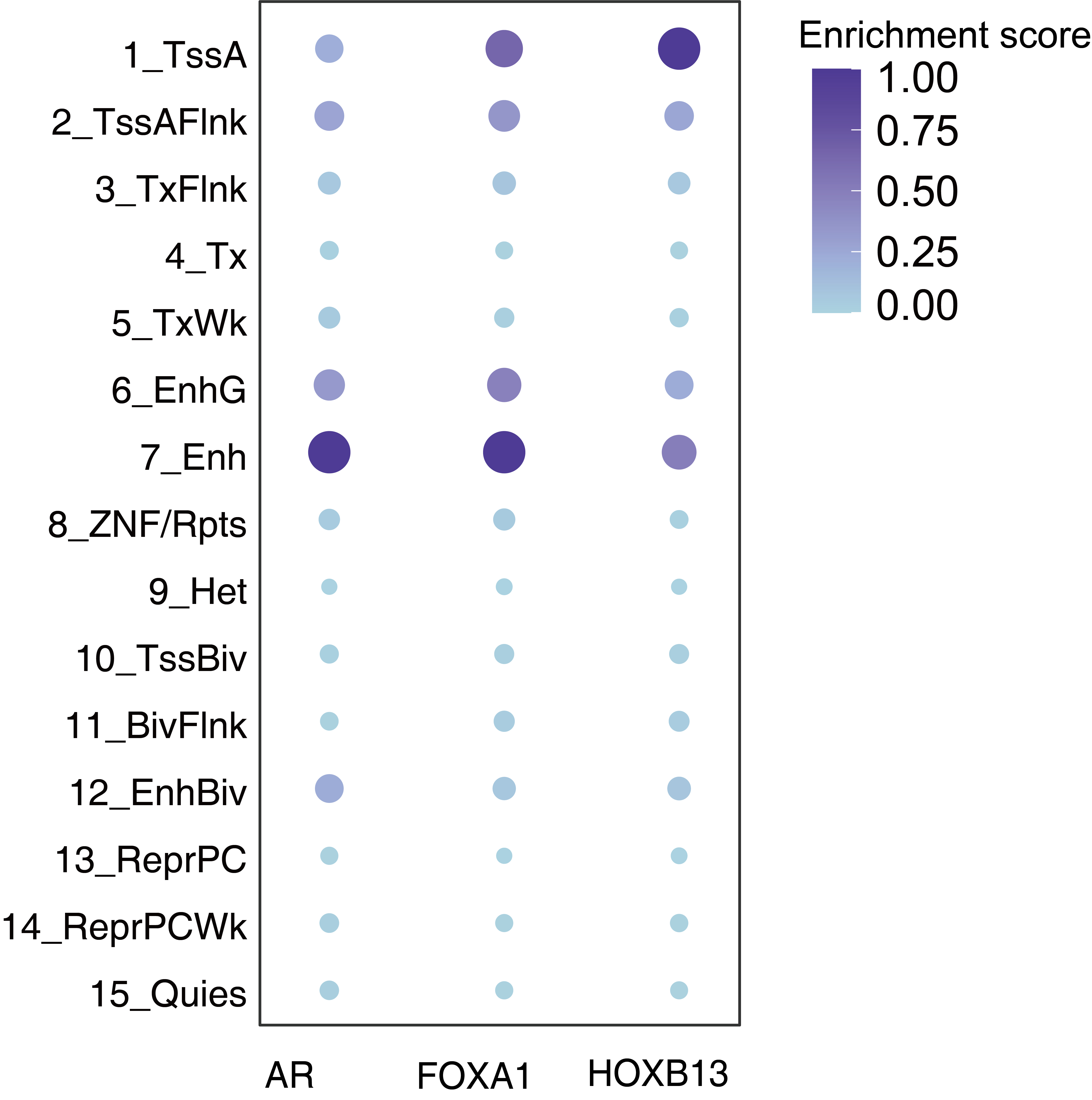

Supplement: Supplementary file 15 [file Image_7.JPEG]

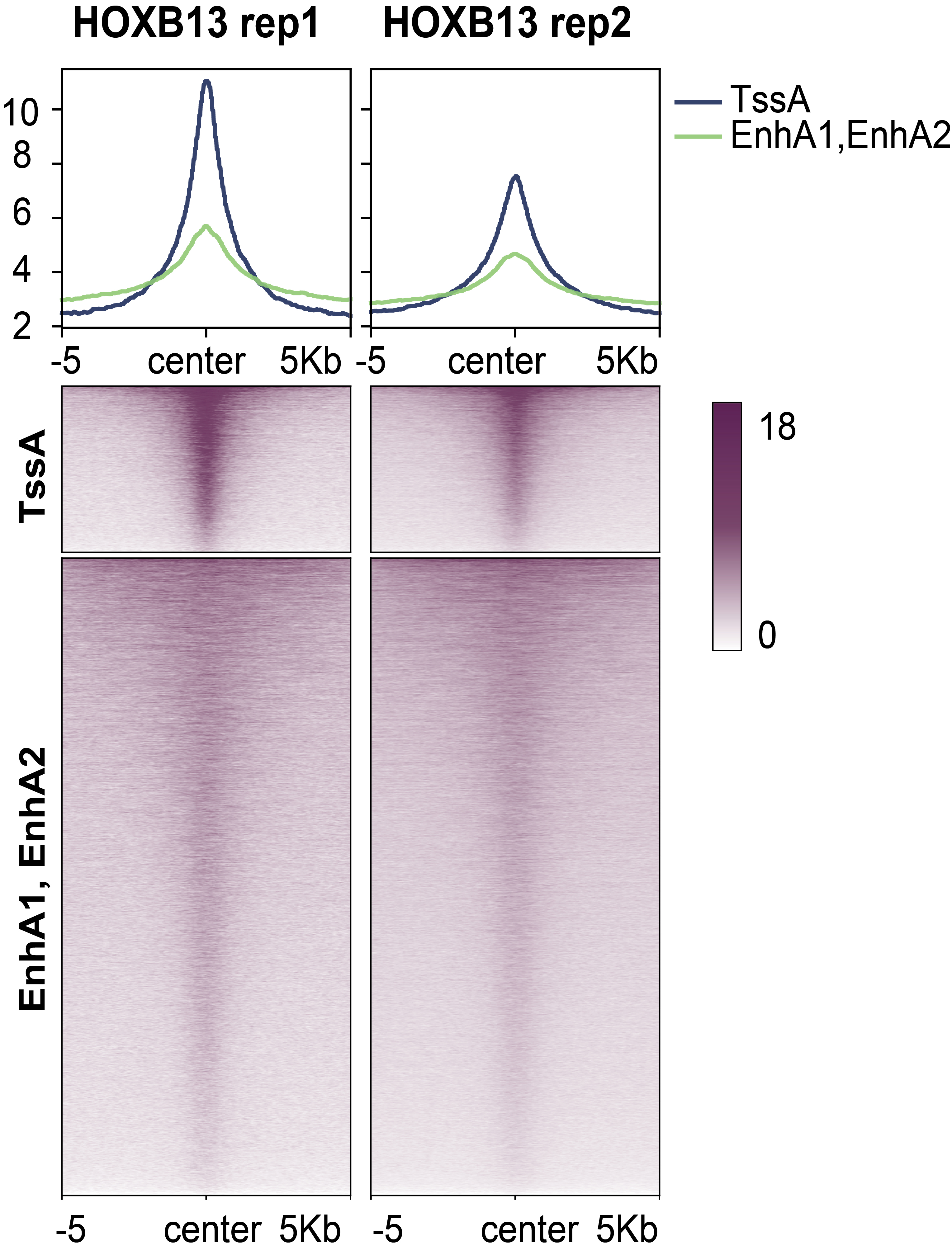

Supplement: Supplementary file 16 [file Image_8.JPEG]
